# Mid-19th century road network dataset for Galicia and Austrian Silesia, Habsburg Empire

**DOI:** 10.1016/j.dib.2019.104854

**Published:** 2019-11-21

**Authors:** Dominik Kaim, Marcin Szwagrzyk, Krzysztof Ostafin

**Affiliations:** Jagiellonian University, Faculty of Geography and Geology, Institute of Geography and Spatial Management, Gronostajowa 7, 30-387 Kraków, Poland

**Keywords:** Road network, Galicia, Austrian Silesia, Habsburg Empire, Historical GIS, HGIS

## Abstract

In this paper, we present the vector dataset of the historical road network of Galicia and Austrian Silesia (>80 000 km^2^) in the mid-19th century – two regions of the former Habsburg Empire, located in Central Europe. The data were acquired manually from 455 map sheets of the Austrian second military survey map (1:28,800) for the four main road categories, according to the map legend. All the road categories present the roads passable at any time of the year, which was strategic information from the military point of view and build a network of 15 461 km. Currently, the data can be used by various researchers studying migrations, regional development, but also human impact on the environment, like land use change, invasive species introduction or landscape fragmentation. The dataset presents the times just before the most dynamic economic changes of the 19th century, which had a great impact on the region. On the other hand, the road network presented here was developed in the conditions of one country, the Habsburg Empire, which collapsed after the First World War, triggering the rise of new states and remodelling the transport network connections in Central Europe. Additionally, the data are accompanied by the layer of towns and villages with more than 2000 inhabitants, based on the 1857 Austrian census data.

**Table:** Specifications Table

Subject	Geography, Planning and Development
Specific subject area	Geography, Geoinformation, History, GIS, Historical GIS
Type of data	Linear vector data layer
How data were acquired	Data acquired by manual vectorisation from the 19th century historical maps.
Data format	Vector data, shapefile
Parameters for data collection	Data were acquired for the 4 main categories of the roads, according to the historical map legend.
Description of data collection	Manual vectorisation of the historical road network of Galicia and Austrian Silesia (>80 000 km^2^) in the mid-19th century – two regions of former Habsburg Empire, located in Central Europe. The data were collected from detailed topographic map sheets of the Austrian second military survey map (1:28,800).
Data source location	Historical regions of Galicia and Austrian Silesia (Central Europe), currently located in Czechia, Poland and Ukraine (>80 000km^2^).
Data accessibility	Repository name: Mendeley Data Data identification number: 10.17632/j9mvw2zwgf.2 Direct URL to data: https://doi.org/10.17632/j9mvw2zwgf.2

**Table:** 

**Value of the Data** • Detailed and consistent dataset on the historical main road network of a large part of Central Europe in the mid-19th century. • Data can be of high importance for social and environmental scientists studying e.g. migrations, accessibility, drivers of regional development, legacies of human impact on the environment like land use change, invasive species introduction, large mammals habitats and many more. • Accessibility is one of the most popular and important spatial variables used in environmental modelling. However, historical road data are missing. This dataset can address this issue. • Roadless areas are now perceived as critical for species connectivity globally; little is known however, about the impact of historical road legacies. • Data were collected based on the detailed 1:28,800 military maps, consistent over a large part of Central Europe and are now available in GIS, shapefile vector format, easy to be used in various spatial analyses, incl. network analysis. • The road network is accompanied by the layer of 309 towns and villages with more than 2000 inhabitants, based on the 1857 Austrian census.

## 1 Data

Evolution of the transportation network plays an important role in development of regions [1] or transformation of the habitats [2]. In the 19th century, the role of the roads started to decrease due to dynamic railway network development [1]. However, the shape of the historical road network is an important indicator of spatial organisation of the area, clearly defining the centres and peripheries [1,3]. In order to analyse it, high-quality historical spatial data are needed. However, due to the time-consuming process of acquisition, they are usually not easily available. The dataset presented here covers four main categories of the roads of the Austrian second military survey maps (1:28,800) from the mid-19th century. As the maps were a result of the cadastral mapping (1:2880) generalisation prepared for military purposes, the categories were defined, taking a strategic military point of view into consideration. It is important to add, that the catalogue of the roads and paths shown on the maps was very expanded, and in this dataset, we included the most important types. The categories were defined from the maps based on [4] and included the roads passable at any time of the year:

1) First class roads (Chaussée, *ger.* Kaiserstrassen I. Klasse)
2) Second class roads (Chaussée, *ger.* Kaiserstrassen II. Klasse)
3) National roads (*ger.* Landstrassen)
4) Maintained roads (*ger.* Erhaltener Fahrwegen)

**Fig. 1:**
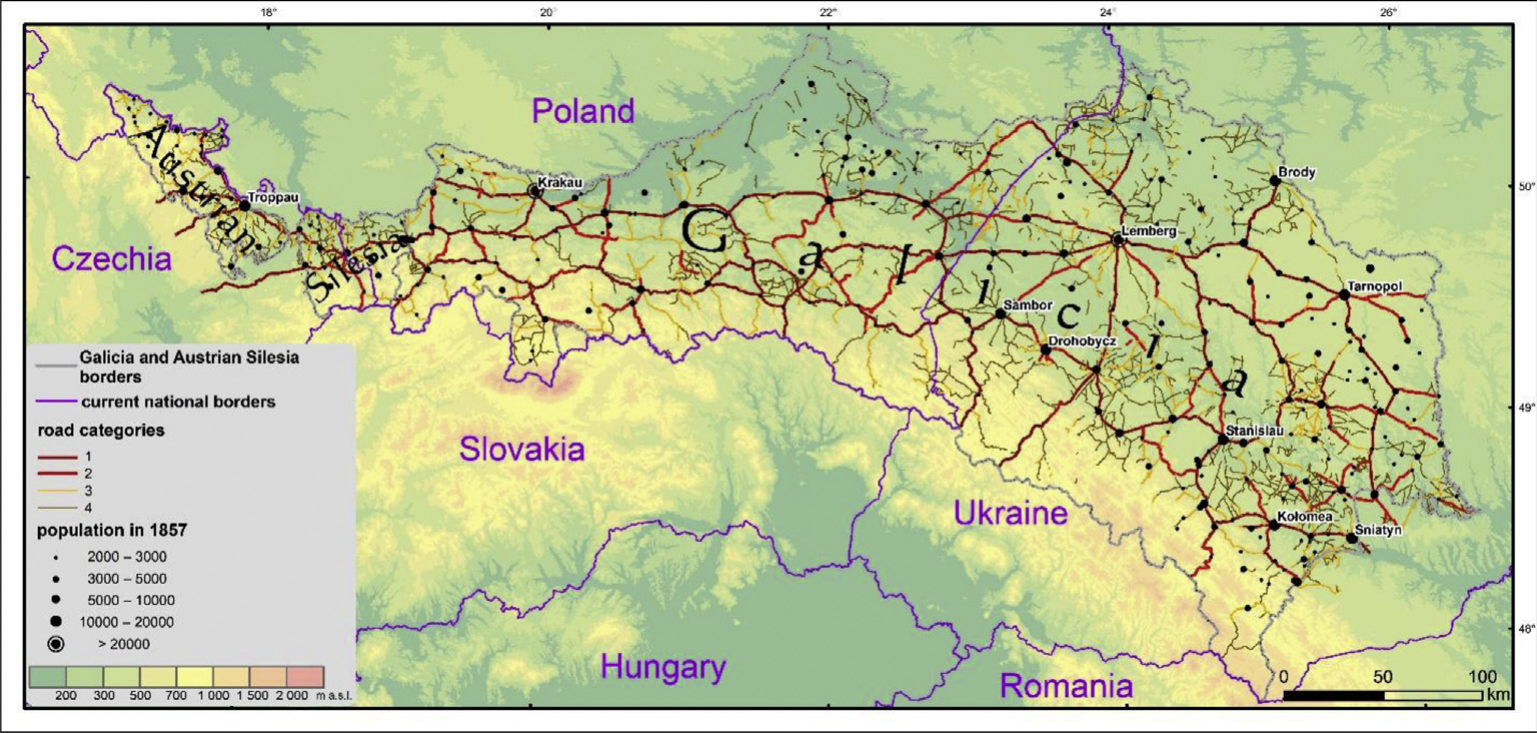
Map of the four main categories of the roads in Galicia and Austrian Silesia, presented on the Austrian second military survey maps. Towns and villages with more than 2000 inhabitants, according to the 1857 census [5].

The first and the second class roads contained the roads built according to the norms assuring usability by heavy military horse carts, all year long, in all weather conditions. First category roads were at least 3 fathoms (*ger.* Klafter) wide, which was equal to ∼5.7 m (1 fathom - 1,8965 m). The second category roads could have less than 3 fathoms [4]. National roads were 16 feet wide (*ger.* Fuss), which was equal to ∼5 m (1 foot - 0,3161 m). Contrary to the first and second class roads, national roads did not have to have ditches, bridges and other protections. Maintained roads had to be passable at any time of the year by the light horse carts, however, they did not have the built layer [4]. Altogether, the network consists of 15 461 km of roads. As the auxiliary data helpful in explaining the shape of the network, we attached the layer presenting the location of 309 towns and villages with more than 2000 inhabitants, according to the 1857 census. The layer includes the names, as presented in the census [5] and contemporary names. The dataset covers the territories located in the current Czechia, Poland and Ukraine (Fig. 1; the data on historical and current boundaries are presented on the Figure to help the readers in correct locating of the dataset and are not included in the described dataset).

## 2 Experimental design, materials, and methods

**Fig. 2:**
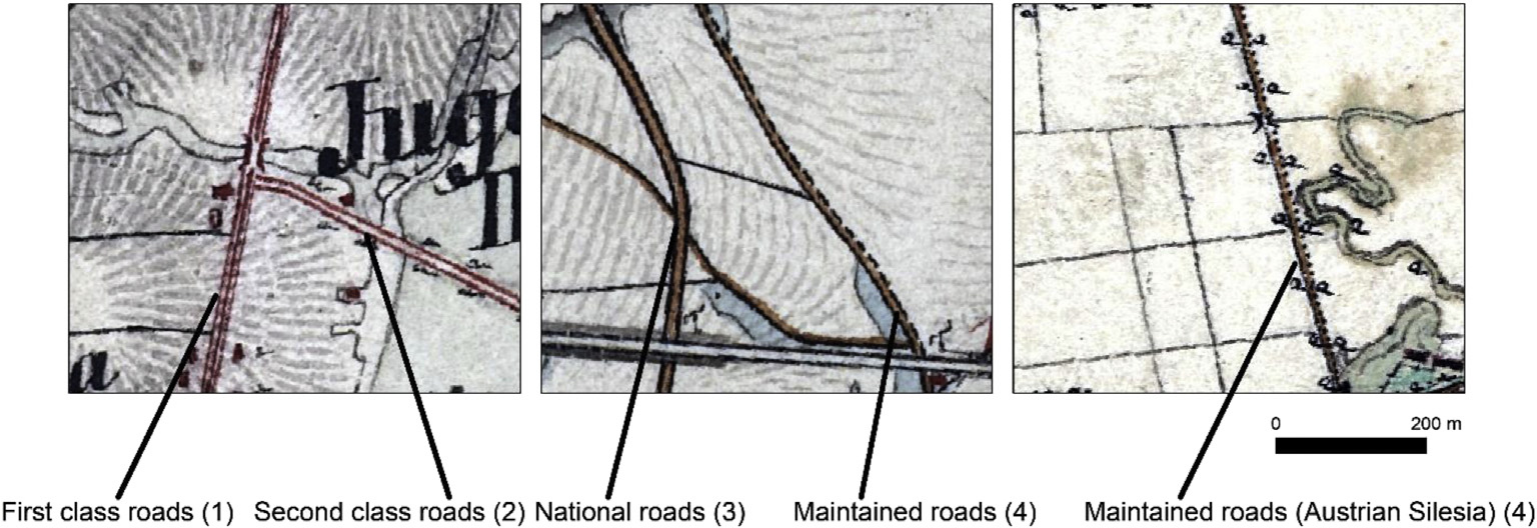
Categories of roads included in the database.

The roads were manually vectorised from the Austrian second military survey maps (1:28,800) i.e., all the roads were re-sketched on-screen in ∼1:5 000 zoom. Before the vectorisation, the maps in the form of 300 dpi tiff files, were georeferenced to the contemporary maps or satellite images, based on 20–30 ground control points per sheet. The control points used in this procedure were stable over time, like historical churches or other buildings, road crossings etc. The RMS error ranged in most cases between 10 and 30 m, with maximal values not exceeding 40 m. The maps covering the Austrian Silesia (42 map sheets) were issued 1837–41, while Galicia (413 map sheets) was mapped 1861–64. Based on the maps, 4 categories of roads, presented in the *Data* section (Fig. 1) were distinguished and vectorised. In the maps covering Austrian Silesia, the category of maintained roads included also the roads marked with the sign presented on the Fig. 2 which was not included in Ref. [4], what might be a result of earlier map edition regarding Galicia, and the time of Zaffauk [4] publication.

**Fig. 3:**
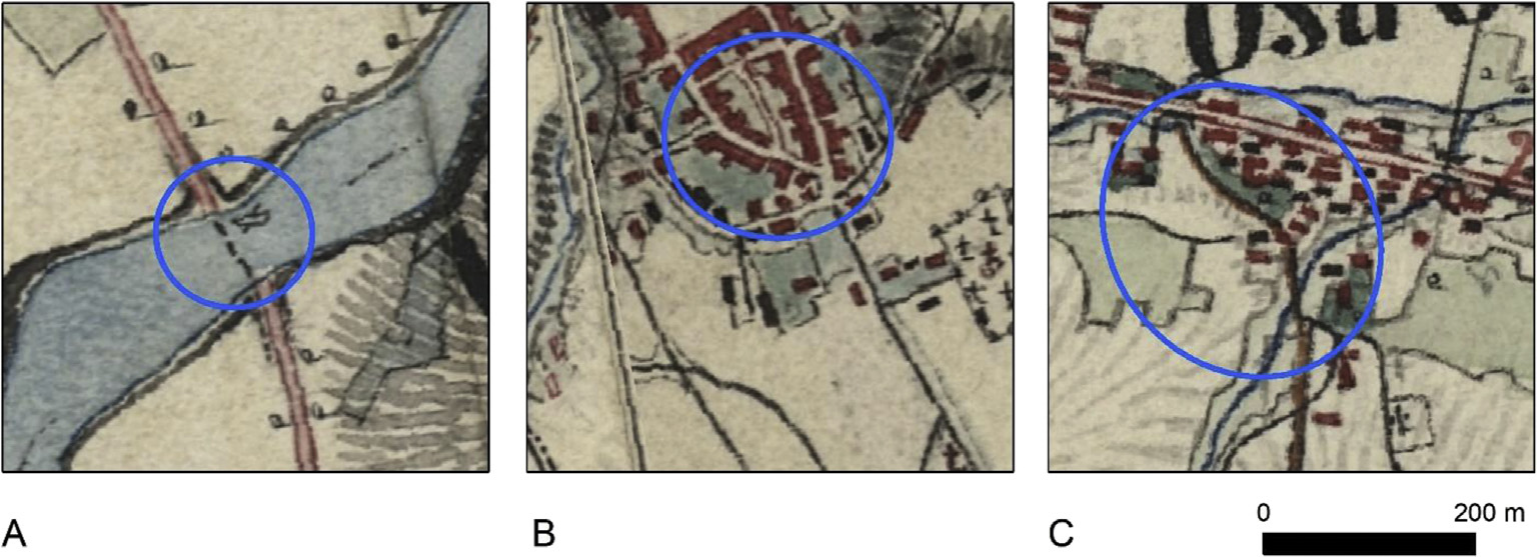
Examples of category no. 5 presented in the database: A – river ferry, B – town roads with no clear category, C – link between second class road and maintained road.

**Fig. 4:**
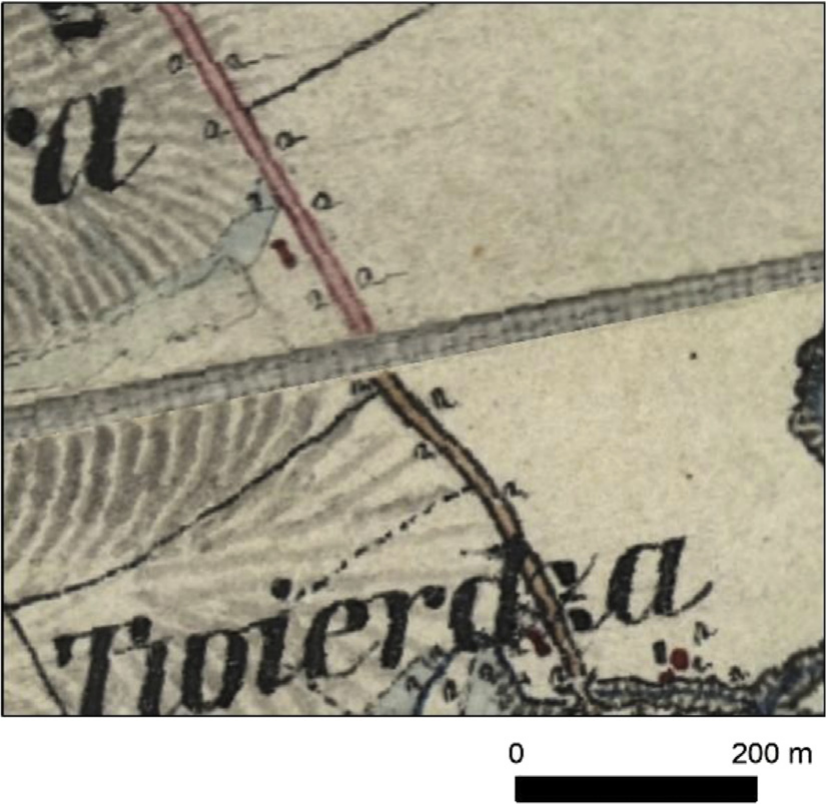
Change of the road category on the neighbouring map sheet from second class road to national road.

Additionally to four categories of main roads, we decided to create also category no. 5, which included – river ferries and river passages on the main roads, main roads in some of the towns, where category was not assigned and short links between the main roads (Fig. 3, Table 1). In some cases, the road category changed with the change of the map sheet (Fig. 4), but as we aimed at creating GIS database, as presented on the original maps, it is reconstructed as it was drawn on the maps. Sometimes the road ends at the edge of the map sheet and does not continue on the neighbouring map, which may be a result of differences in road classification, done by the military cartographers. In such cases, it is presented in the database in the same categories, as on the original map. In order to assure the high quality of the data, we used snapping tools during data acquisition, what helped in connecting the roads topologically correct, and resulted in creation a network, when possible. Additionally, the network topology was verified in ArcMap topology tools by a set of rules: *Invalid Geometry Check, Geometry on Geometry Checks (Overlaps), Orphan Check.* Due to the nature of the data, the orphans (sections not connected to the rest of the dataset), where found mainly in the 3–5 road categories, while in the 1–2 road categories, only 3 sections were not connected (which was in line with the original map content). Two other, above-mentioned topology rules indicated no errors in the dataset. Dataset can be used in network analysis, like best route, creating service areas etc.

**Table 1:** Length of the road categories in the database; * - other categories include e.g. passages, links and selected road networks in the towns; full explanation can be found in the manuscript.

Road category	name	Length [km]	% of the network
1	First class roads	2814	18.2
2	Second class roads	1502	9.7
3	National roads	3542	22.9
4	Maintained roads	7419	48.0
5	Other*	184	1.2
	sum	15 461	100

Finally, the network covers 15 461 km (Table 1) and, to make the network consistent, includes also the section of the First Class Road going through Moravia, located between the Austrian Silesia and Galicia.
